# Origin and physical effects of edge states in two-dimensional Ruddlesden-Popper perovskites

**DOI:** 10.1016/j.isci.2022.104420

**Published:** 2022-05-18

**Authors:** Junlin Lu, Chunhua Zhou, Weijian Chen, Xin Wang, Baohua Jia, Xiaoming Wen

**Affiliations:** 1 Centre for Translational Atomaterials, Swinburne University of Technology, Hawthorn VIC 3122, Australia; 2 College of Physics and Optoelectronics, Key Lab of Advanced Transducers and Intelligent Control System of Ministry of Education, Taiyuan University of Technology, Taiyuan, Shanxi 030024 China; 3 Australian Centre for Advanced Photovoltaics, School of Photovoltaic and Renewable Energy Engineering, University of New South Wales (UNSW), Kensington, NSW 2052, Australia; 4 South China Academy of Advanced Optoelectronics and International Academy of Optoelectronics at Zhaoqing, South China Normal University, Zhaoqing, Guangdong 510631, China; 5 Guangdong Provincial Key Laboratory of Optical Information Materials and Technology, Institute of Electronic Paper Displays, South China Academy of Advanced Optoelectronics, South China Normal University, Guangzhou, Guangdong 510006 China; 6 School of Science, RMIT University, Melbourne, VIC 3000, Australia

**Keywords:** Transport phenomena, Materials science, Nanomaterials

## Abstract

The edge region of two-dimensional (2D) Ruddlesden-Popper (RP) perovskites exhibits anomalous properties from the bulk region, including low energy emission and superior capability of dissociating exciton, which is highly beneficial for the optoelectronic devices like solar cells and photodetectors, denoted as “edge states”. In this review, we introduce the recent progress on the edge states that have been focused on the origin and the optoelectronic properties of edge states in 2D RP perovskites. By providing theoretical frameworks and experimental observations, we elucidate the origin of the edge states from two aspects, intrinsic electronic properties and extrinsic structure distortions. Besides, we introduce the physical properties of the edge states and current debating on this topic. Finally, we present an outlook on future research about the edge states of 2D RP perovskites.

## Graphical abstract

**Figure:**
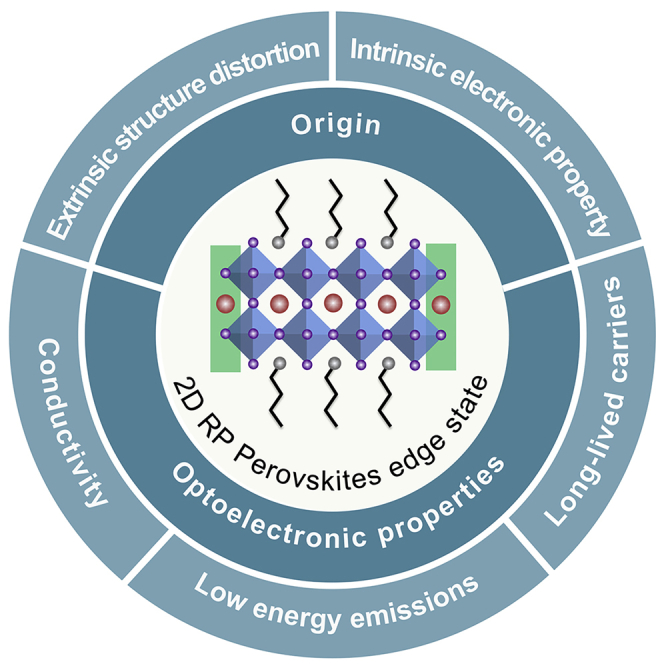

## Introduction

In recent years, two-dimensional (2D) Ruddlesden-Popper (RP) perovskites have become one of the hottest research topics due to their excellent environmental stability together with the unique optoelectronic properties which have shown a great potential for solar cells (Cao et al., 2015; Chen et al., 2020; Gharibzadeh et al., 2019; Grancini et al., 2017; Kim et al., 2020; Krishna et al., 2019; Liu et al., 2019; Wang et al., 2017, 2018, 2019a; Zhang et al., 2019a; Zuo et al., 2019) and light emitters (Kong et al., 2021; Quan et al., 2018; Tsai et al., 2018; Vashishtha et al., 2019; Yuan et al., 2016; Zhao et al., 2018). The typical composition of 2D Ruddlesden-Popper perovskites is (RNH_3_)_2_A_n−1_M_n_X_3n+1_, where the RNH_3_ acts as spacer cation, which is aromatic or long-chain alkylammonium cation, such as phenylethylammonium cations (PEA^+^) and bultyammonium cations (BA^+^); A is a small organic cation, like methylammonium cations (MA^+^) and formamidinium cations (FA^+^); M is a divalent metal cation such as Pb^2+^ and Sn^2+^; X is halide anion, typically bromide and iodine; n is the number of the [MX_6_]^4-^ octahedral layers (Chen et al., 2018). For concise expression, we use the layer number *n* to represent the thickness of 2D RP perovskites in the following section. As the number of [MX_6_]^4-^ octahedral layers decreases from infinity for 3D perovskites to a lower number for 2D RP perovskites, the quantum and dielectric confinement effect increases (Blancon et al., 2017, 2018, 2020; Kamminga et al., 2016; Katan et al., 2019; Leng et al., 2018, 2020; Mauck and Tisdale, 2019; Stoumpos et al., 2016; Tian et al., 2020; Yin et al., 2017), leading to the increased binding energy, ranging from few tens of meV to a few hundred meV (Ahmad et al., 2013; Blancon et al., 2018; Deng et al., 2020; Dyksik et al., 2021; Gauthron et al., 2010; Gelvez-Rueda et al., 2017; Miyata et al., 2015; Saba et al., 2016; Scholz et al., 2018; Spitha et al., 2020; Urban et al., 2020; Yang et al., 2015; Yangui et al., 2015). Besides, the hydrophobic spacer cation enhances the moisture resistance of 2D RP perovskites and interlaminar van der Waals interactions, which makes 2D RP perovskites more stable under ambient environment (Quan et al., 2016; Tsai et al., 2016).

Recently, the edge states of 2D RP perovskites have attracted extensive attention due to their unique properties like emission at lower energy, superior ability to dissociate excitons into long-lived free carriers, and higher conductivity (Blancon et al., 2017; Wang et al., 2019b, 2020; Zhao et al., 2019). When the photogenerated excitons diffuse from bulk to edge region, the excitons dissociate into long-lived free carriers, leading to low energy emissions and conductivity at the crystal edge (Blancon et al., 2017; Feng et al., 2018; Zhao et al., 2020). The behavior of excitons and/or free carriers is intimately correlated with the optoelectronic properties and thus their applications. For solar cells and photodetectors, free carriers are favorable than excitons because they lead to an improved charge extraction efficiency and enhances the performance of the devices. But for LED, excitons are desirable than free carriers because LED requires efficient radiative electron-hole recombination and the free carriers will reduce the quantum efficiency of LED. Thus, it is essential to consider exciton dissociation and free carrier property of 2D RP perovskites when designing optoelectronic devices (Baranowski and Plochocka, 2020; Baranowski et al., 2019; Blancon et al., 2017; Buizza et al., 2019; Cho et al., 2020; Fujiwara et al., 2020; Gan et al., 2021; He et al., 2020; Kinigstein et al., 2020; Li et al., 2020a, 2020b; Lin et al., 2020; Magdaleno et al., 2021; Milot et al., 2016; Motti et al., 2019; Proppe et al., 2018; Zhang et al., 2019b, 2020; Zhou et al., 2019, 2020).

It has been confirmed that the binding energy of 2D RP perovskites is approximately from 450 meV to 100 meV for *n* = 1 to *n* = 5, respectively (Blancon et al., 2018; Dyksik et al., 2021; Gelvez-Rueda et al., 2017), which suggests that exciton, rather than free carrier, should be dominant. However, free carriers have been observed in the low *n* (*n* ≥ 2) 2D RP perovskites, contradicting the conventional concept (Blancon et al., 2017; Feng et al., 2018; Lu et al., 2021; Zhao et al., 2019, 2020). Despite the large binding energy and strong quantum confinement effect, the photogenerated excitons in such 2D perovskites with a small number of layers can be effectively dissociated into free carriers by the edge states in the 2D RP perovskites. This unique property of the edge states can effectively improve the performance of the 2D perovskite-based solar cells and photodetectors. Blancon et al. (Blancon et al. 2017) fabricated solar cells based on 2D RP perovskites and power conversion efficiency (PCE) of 12% were achieved with the assistance of the edge states. In contrast, PCE of the solar cells fabricated by the 2D RP perovskites without the edge states exhibited a dramatic decrease, which was about 1% for *n* = 1 and 2% for *n* = 2. They proposed that the edge states are highly beneficial for the performance of solar cells from two aspects, the extended visible light-harvesting range of the solar cells and the efficient charge carrier collection due to efficient excitons dissociation into free carriers. This emphasizes the important role of edge states in the performance of solar cells. Feng and co-workers (Feng et al., 2018; Zhao et al., 2020) fabricated successively ultrasensitive photodetectors based on 2D RP perovskite nanowire array. They suggested that the ultrasensitivity is attributed to the free carriers in the nanowire array which originates from the effective exciton dissociation by the edge states at the surface of nanowires. Hence, it is critically important to fully understand the edge states of 2D perovskites and further control the edge states for improving the performance of optoelectronic devices.

Inspired by such intriguing nature of edge states, the effects of the edge states have been intensively investigated theoretically and experimentally, mostly focusing on the origin of the edge states and their physical effects in optoelectronic applications. At present, the proposed origin of the edge states can be classified into two theories. One considers that the edge states are the intrinsic electronic states at the edge that makes the bandgap of the edge area narrower than that of the bulk area (Hong et al., 2021; Kepenekian et al., 2018; Wang et al., 2020; Zhang et al., 2019b). Another is that the extrinsic structure distortion at the edge leads to lower energy emissions (Kinigstein et al., 2020; Qin et al., 2020; Shi et al., 2019; Zhao et al., 2019). Supported by significant theoretical and experimental evidence for each of the viewpoints, however, each theory cannot fully interpret all aspects of the observations. More importantly, the experimental results reported in these works contradict each other. It has been consistently confirmed that pristine perovskites with layer number *n* = 1 do not exhibit the effect of edge states (Blancon et al., 2017; Qin et al., 2020; Shi et al., 2019; Zhao et al., 2019). However, the anomalous conductivity in the edge, which is ascribed to the edge states, was observed on the terrace edge of *n* = 1 perovskites (Wang et al., 2019b, 2020). Qin et al. (Qin et al. 2020) suggested that the self-form 3D perovskites on the edge of 2D perovskites are the origin of the edge states, while Shi et al. (Shi et al. 2019) considered the edge state does not belong to their 3D counterpart by observing the crystal lattice distortion at the edge of (BA)_2_FAPb_2_I_7_. To date, the consensus has not yet been reached on the origin of the edge states in 2D perovskites and relevant physical effects. A systematic review on the edge states in 2D perovskites is urgently demanded to provide a comprehensive understanding of it.

In this review, we summarized the most up-to-date research progress of edge states on 2D RP perovskites. In the first part, we review the different optoelectronic properties of the 2D RP perovskiteedge states like carrier dynamics and PL behavior. In the second part, we provide a detailed discussion on the origin of the edge states, including the density functional theory (DFT) calculations of the edge states and the experimental evidence. Last but not least, we summarize and discuss the current contradictory experimental results on edge states and provide an outlook on future research. In order to precisely interpret the concept of edge states in 2D halide perovskites to avoid confusion about this term in other disciplines, here we define the edge states as the narrower band gap formed near the edge region of the 2D RP perovskites crystal than that in the bulk region, in which the low energy emission or different conductivities is generally observed.

## Physical properties of edge states

**Figure 1:**
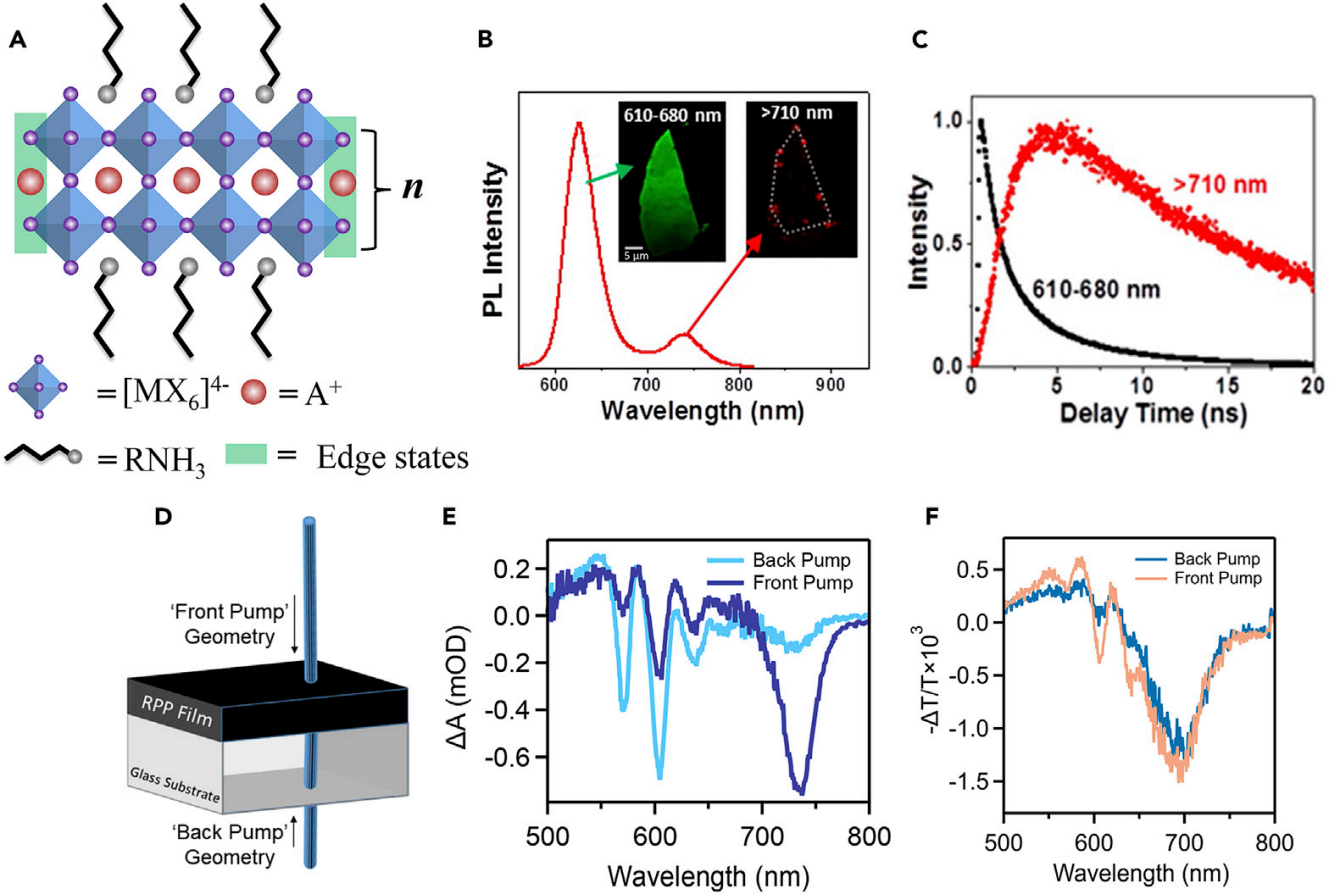
The 2D RP perovskite structure and the optical properties of the edge states (A) Schematic of 2D perovskite structure. (B) Microscopic PL spectra of the (BA)_2_(MA)_2_Pb_3_I_10_ (*n* = 3) exfoliated 2D crystal. The insert is the PL images collected at emission channels of 610–680 (interior) and >710 nm (crystal edges). Scale bar: 5 μm. (C) The corresponding time-resolved PL kinetics of (BA)_2_(MA)_2_Pb_3_I_10_ collected in the emission channels of 610–680 (interior) and >710 nm (crystal edges). Figures 1B and 1C adapted from (Zhao et al., 2019). Copyright © 2019, American Chemical Society. (D–F) Schematic of the front/back pumping geometry transient absorption characterization for perovskite films and the 5 ps transient spectra obtained in different geometries with (BA)_2_(MA)_2_Pb_3_I_10_ hot casting (E) and post annealed (F) film, reprinted from (Kinigstein et al., 2020). Copyright © 2020, American Chemical Society.

The general crystal structure of 2D RP perovskite is shown in Figure 1A: the inorganic octahedral connects in corner-sharing configuration to form an inorganic layer; the larger organic spacer cation connects with the inorganic layer via hydrogen bonds and separates the different inorganic layers (Stoumpos et al., 2016). For ideal crystals, the crystal structure is consistently repeated without boundaries. In reality, however, the structure of a crystal would have inevitable boundaries. The chemical circumstances of the atoms at the edge of the crystal differ from those within the bulk, which makes the edge region of the material exhibit different properties than the bulk. For example, the different edge configurations have been confirmed to significantly impact the optoelectronic properties of the typical 2D material of graphene (Plotnik et al., 2014; Wang et al., 2016).

As for 2D RP perovskites, their edge regions have been reported to exhibit many unique properties different from the bulk region. The most characteristic property of the edge states is the low energy PL emission. The edge state was first observed on the (BA)_2_(MA)_n-1_Pb_n_I_3n+1_ with *n* > 2 (Blancon et al., 2017). By PL mapping with multiple spectral bands, the lower energy emission was observed exclusively from the edge (1.68eV) in an exfoliated crystal of (BA)_2_(MA)_2_Pb_3_I_10_ (*n* = 3), in addition to the conventional emission band at 2.01eV from all over the crystal (Figure 1B) (Blancon et al., 2017; Zhao et al., 2019). When using time-resolved PL spectroscopy to analyze these PL emissions, the edge states exhibit a significantly slower PL decay, with a lifetime as long as four times of the bulk emission. Moreover, the PL emissions of edge states exhibit a significantly slower rise time. This suggests the charge carriers spend a very long time, in nanoseconds, to reach their population maximum than the case of bulk (Figure 1C), indicating the carrier transport from the bulk region to the low energy edge states (Blancon et al., 2017; Liang et al., 2021; Shi et al., 2019; Zhao et al., 2019). The lower PL energy and the longer PL lifetime of edge states suggest that edge states provide a direct pathway for dissociating the photogenerated excitons into free carriers even though the average binding energy at a level of a few hundred meV for *n* ≥ 2.

The edge state emission was also observed in 2D RP perovskite films (Blancon et al., 2017; Kinigstein et al., 2020). But different from the exfoliated crystal which exhibits two PL peaks, the PL spectra of 2D RP perovskite films were dominated by the low energy emission of the edge states and exhibited only one PL peak at low energy region, implying the edge states are strongly located at the surface of films (Blancon et al., 2017; Kinigstein et al., 2020). This observation can be further confirmed by ultrafast transient absorption (TA) spectroscopy. The films exhibited different TA spectra when the pump was incident from the front and rear sides, indicating the asymmetric edge state concentration distribution was along with the depth of the films (Figures 1D and 1E) (Kinigstein et al., 2020). It was also found that the fabricated method will affect the distribution of the edge states; the TA spectra of post annealed films exhibit less asymmetry in front/rear side incidents compared with that of hot casting films (Figure 1F) (Kinigstein et al., 2020). The dominant low energy emission from the edge states can be also observed on the 2D RP perovskite nanowires, indicating the edge states are similarly located at the surface of the nanowires (Feng et al., 2018; Zhao et al., 2020).

**Figure 2:**
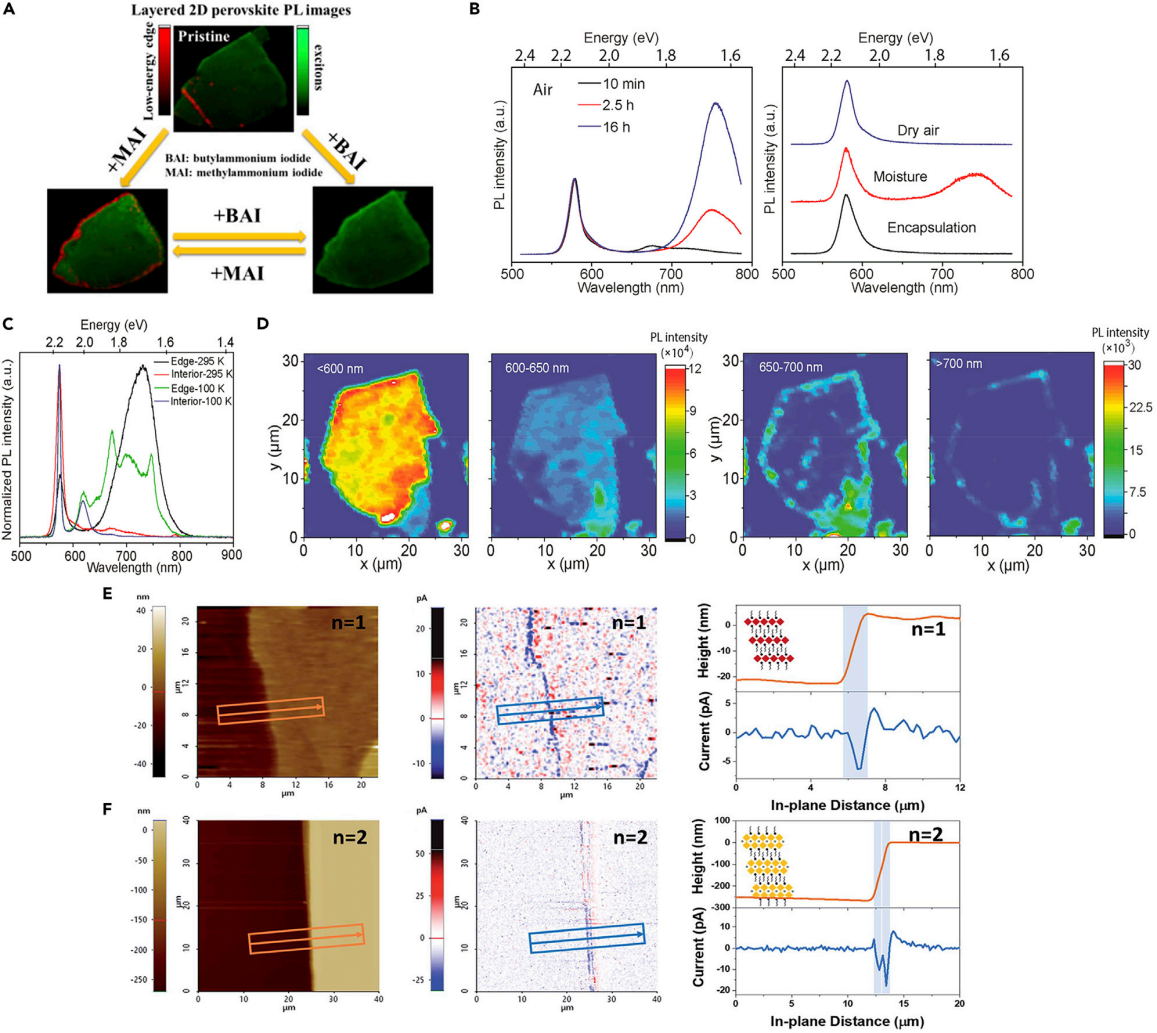
Controlling the edge state emission and the conductivity of the edge states (A) Schematic of the controlled edge state emission by MA/BA cation exchange process. Reprinted from (Zhao et al., 2019). Copyright © 2019, American Chemical Society. (B) PL spectra of the edge of an exfoliated BA_2_FAPb_2_I_7_ crystal. Left panel: with different time exposed to air; Right panel: with different storage atmosphere. (C) PL spectra of the BA_2_FAPb_2_I_7_ crystal edges and interior regions at room and low temperature. (D) PL mapping of exfoliated BA_2_FAPb_2_I_7_ crystal at 100 K with different emission ranges. Figures 2B–2D reprinted from (Shi et al., 2019). Copyright © 2019, American Chemical Society. (E and F) The CGM results of (BA)_2_PbI_4_ (*n* = 1) and (BA)_2_(MA)Pb_2_I_7_ (*n* = 2). Left panel: The topographical images; middle panel: the current images; Right panel: The corresponding average line profile measured along the arrow direction from the topographical and current images. Reprinted from (Wang et al., 2020). Copyright © 2020, Royal Society of Chemistry.

The emission of the edge states in 2D RP perovskites has shown controllable nature by the post-treatment process. Zhao et al. (Zhao et al. 2019) found that the edge state emission of (BA)_2_(MA)_n−1_Pb_n_I_3n+1_ can be controlled by an MA^+^/BA^+^ cation exchange process, removing emission by rinsing with BAI solution and generating by rinsing with MAI solution, and this cation exchange process is repeatable (Figure 2A). Even though the edge state emission is absent for *n* = 1 RP perovskite, the edge state emission can be generated after the MA cation exchange treatment. Moreover, the edge states of (BA)_2_FAPb_2_I_7_ and (BA)_2_MA_2_Pb_3_Br_10_ can also be generated by exposing the sample to the moisture condition even though the moisture concentration is as low as 0.5 ppm; and the intensity of edge state emission increases with increasing exposure time to moisture (Figure 2B) (Shi et al., 2019). Except for post-treatment, the chemical composition also strongly affects the edge states. For 2D RP perovskites with *n* = 2, the edge state emission of (BA)_2_MAPb_2_I_7_ is absent in some case, but the edge state emission certainly occurs when the A-site cation changes from MA^+^ to FA^+^ ((BA)_2_FAPb_2_I_7_), which can be attributed to the larger size of FA^+^ cation inducing extra strain at the edge than the case in MA^+^ (Shi et al., 2019). Besides, recent research shows that the peak of edge state emission exhibits less redshift when the ligand is changed from n-butylamine to n-pentylamine for (RNH_3_)_2_(MA)Pb_2_Br_7_ (RNH_3_ represents ligand) (Liang et al., 2021). The shift of the PL peak of edge state emission can also be controlled by the thickness of the single crystal; the redshift of edge state emission increases with the increasing thickness of the single crystal (Shi et al., 2019).

The behavior of the edge states at low temperatures has also been studied. Shi et al. (Shi et al. 2019) found that the edge state emission exhibits multiple peaks at low temperature, in sharp contrast to the single edge state emission peak at room temperature. When the temperature decreased to 100 K, the edge state emission of exfoliated BA_2_FAPb_2_I_7_ crystal exhibited multiple peaks located at 620, 672, 700, and 746 nm (Figure 2C). When further using PL mapping technique to monitor the multiple edge state emission peaks (Figure 2D) at 100 K, the PL emission above 650 nm was restricted to the crystal edge, while the PL emission within 600–650 nm was evenly dispersed throughout the crystal. This result suggests that the edge states are complex combinations of several low-energy states.

The conductivity is another anomalous property of the edge states in 2D RP perovskites. By using conducting atomic force microscopy (c-AFM) and charge gradient microscopy (CGM), Wang et al. (Wang et al., 2019b, 2020) observed that the exfoliated (BA)_2_(MA)_n–1_Pb_n_I_3n+1_ exhibited distinctive conductivity at crystal edge, while the bulk region was insulative. As shown in Figures 2E and 2F, no discernible current signal was detected from the bulk region of the crystal, but a sharp current signal was confirmed when the tip of AFM moved to the edge region of the crystal, indicating the conductivity of the edge region of 2D RP perovskites. The distinctive conductivity can be ascribed to the edge states which can efficiently dissociate excitons into free charge carriers and lead to separated electron and hole and therefore an increased charge density at the edge than that at the bulk region. They also found that the current magnitude at the edge was only related to the height of the crystal edge, which suggested a constant carrier density at the edge, about 7.5 × 10^−3^ pC nm^−1^. It is noteworthy that the electric-conducting feature was also observed in the exfoliated crystal with *n* = 1, which obviously contradicts the optical observation.

## Origin of the edge states

As discussed in the above section, the edge states provide unique properties for 2D RP perovskites like free carrier property and conductivity. What’s more, some properties of edge states are controllable, allowing broad prospects for different applications in the future. It is critically important to have a comprehensive understanding of the edge states for future applications of 2D RP perovskites, and figuring out the origin of the edge states is the key point. At present, numerous articles have been published on the origin of the edge states; but there still lacks a unified conclusion. In the following section, up-to-date research progress will be discussed in detail to provide a comprehensive understanding of the fundamental origin of the edge states in 2D RP perovskites.

### The intrinsic property theory

**Figure 3:**
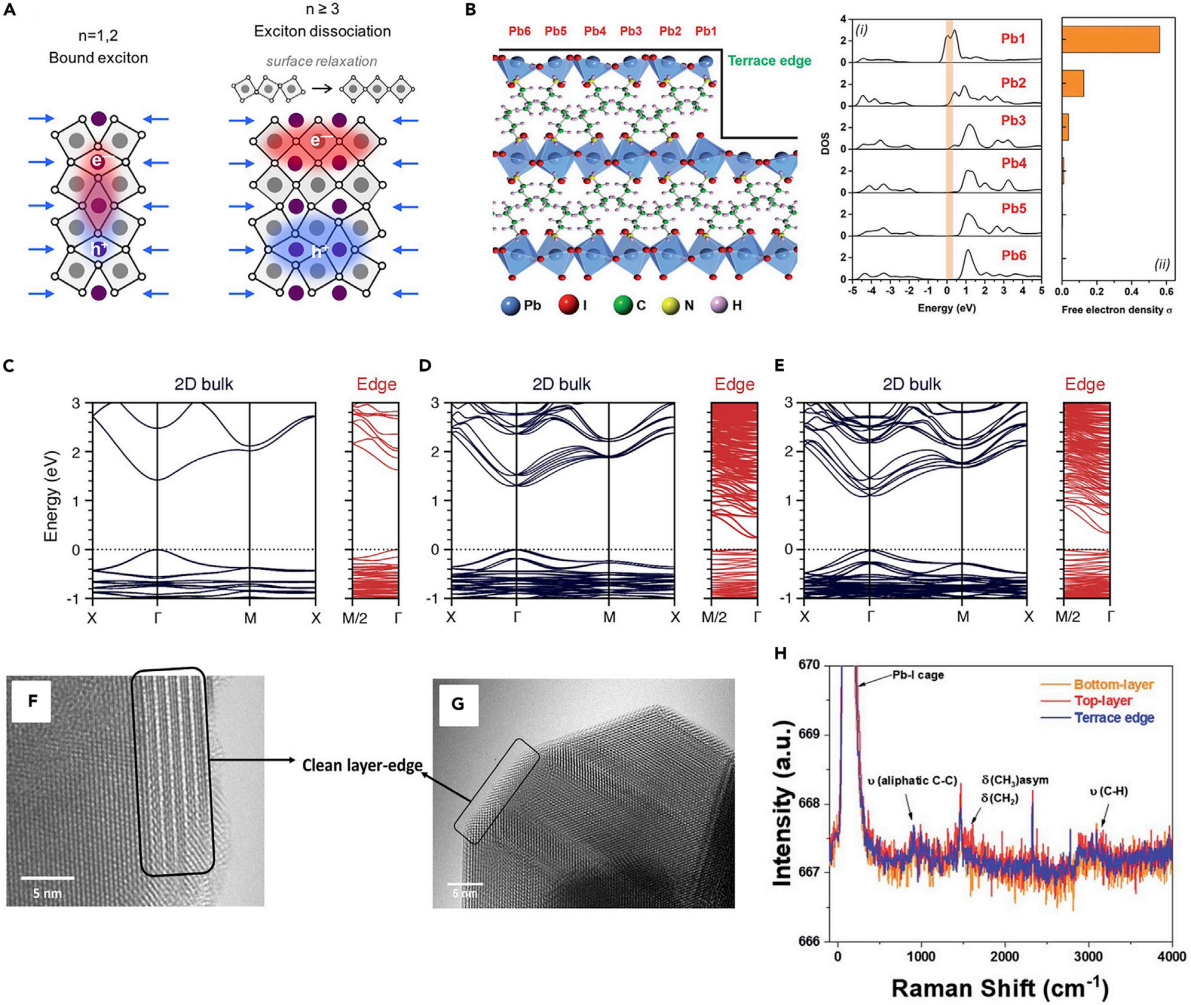
The intrinsic property theory (A) Schematics of the surface-induced exciton dissociation in 2D RP perovskites with *n* ⩾3, Reprinted from (Kepenekian et al., 2018). Copyright © 2018, American Chemical Society. (B) Left panel: Schematic lattice of a surface-BA_2_PbI_4_ supercell constructed for first-principles calculations; Right panel:(i) Density of states (DOS) profiles and free-electron densities of the Pb–I planes in surface BA_2_PbI_4_, (ii) Corresponding free electron density calculated from the DOS near the Fermi level (0–0.3 eV). Reprinted from (Wang et al., 2020). Copyright © 2020, Royal Society of Chemistry. (C–E) Band structures of 2D bulk and edges of (BA)_2_(MA)_n−1_Pb_n_I_3n+1_ for *n* = 1, *n* = 2, and *n* = 3, respectively, Reprinted from (Hong et al., 2021). Copyright © 2021, American Chemical Society. (F and G) High-resolution transmission electron microscopy (HRTEM) images of BA_2_MA_3_Pb_4_I_13_ single-crystal layer-edge which shows clean layer edges. (H) Raman spectra obtained from different regions fo the BA_2_MA_3_Pb_4_I_13_ single crystal. Figures 3F–3H reprinted from (Wang et al., 2020). Copyright © 2020, Royal Society of Chemistry.

When the edge states were first reported by Blancon et al., it was supposed that the lower energy emission and the longer carrier lifetime originates from the intrinsic electronic structure at the edge of the 2D perovskites, i.e., distortion of the octahedra, exciton self-trapping, or dangling bonds (Blancon et al., 2017). Later in 2018, Blancon and co-workers published the first theoretical article about the origin of edge states. They proposed that the edge states were the consequence of the lattice mismatch (Kepenekian et al., 2018). For 2D layered perovskites of *n* > 2, due to the rotational degrees of freedom, the surface octahedral layer expansion would be eased by the contraction of the subsurface octahedral layer, leading to the decoupling of the top surface octahedron layer from the subsurface ones, thus relaxing the internal elastic energy on the surface. In contrast, the surface octahedra layers of *n* = 1 and 2 have scarcely rotational degrees of freedom; the octahedral layer expansion would be observed in the whole surface region and the surface relaxation process would not occur. The different surface relaxation processes led to a different surface arrangement and also changed the bandgap potential. For *n* = 1 and *n* = 2, the surface bandgap became wider than the bandgap of the bulk region, while for *n* = 3 and *n* = 4, the surface bandgap became narrower than the bandgap of the bulk region. The surface octahedral relaxation was supposed to be the reason for the narrower bandgap of *n* = 3 and 4 due to the appearance of in-gap electronic states. Besides, according to the localized density of states calculation, for *n* > 2, the carrier was separated due to the in-gap electronic states and the electrons were mainly localized at the top of the octahedron layer, exhibiting free carrier characteristic; however, for *n* = 1 and 2, the charge dispersed evenly through the whole region, exhibiting exciton characteristic (Figure 3A). In a word, the different surface relaxation processes between *n* = 1, 2 and *n* > 2 resulted in the different band alignment and charge separation. In the case of *n* > 2, the subsurface lattice compression resulted in the in-gap electronic states, leading to the redshift of bandgap, which was supposed to be the major reason for the edge states.

Soon after, Zhang et al. (Zhang et al. 2019b) performed a theoretical investigation on the origin of the edge states and they argued that the edge states were the result of the different chemical properties between I and Pb atoms. The unsaturated iodine bonds at the edge were the main reason for the charge separation of the edge states. In 2D perovskites, the valence band maxima (VBM) primarily consisted of I atomic orbitals while the conduction band minima (CBM) was formed by Pb atoms orbitals. The unsaturated iodine and lead bonds at the edge region would not create trap states within the original bandgap but it still affected the orbital localization. The metallic Pb atom could eliminate unsaturated chemical bonds and heal defects by changing their oxidation states, which makes the electrons more delocalized. While, the unsaturated covalent bonds of iodide provided a strong force to localize the holes. This asymmetry confinement effect between electron and hole led to the separation of charges, resulting in the edge states. In addition, the long-lived carriers in the edge states can be explained by the small overlapping of wave function between the electron and hole and the short quantum coherence between excited and ground electronic states.

Wang et al. (Wang et al. 2020) proposed that the unusual electronic property of the Pb atom at the edge was the reason for the edge state. According to the density of states (DOS) calculation based on the terrace edge model, the Pb atom at the edge surface had a remarkable higher free electron density than other Pb atoms which were away from the edge surface (Figure 3B), leading to the conductivity of the terrace edge. Besides, the conduction band of the edge region would shift downward to form lower energy states than that of the bulk region, forming a narrower bandgap at the edge region, resulting in the low-energy PL emission at the edge.

Furthermore, another theoretical hypothesis proposed that the reason for the edge states was the internal electric fields induced by the polarized molecular alignment of A-site cation, providing a different understanding of intrinsic electronic structure (Hong et al., 2021). The space group of *n* ≥ 2 can be divided into two different structures according to the orientational of A-site cation, centrosymmetric or noncentrosymmetric, and the noncentrosymmetric structure was more energetically stable than the centrosymmetric structure. Meanwhile, the space group of *n* = 1 only had one centrosymmetric structure due to the lack of the orientable A-site cation. In a noncentrosymmetric structure, the neighboring MA cations pointed to the same directions parallel to the octahedral layers that create a finite dipole moment, leading to the in-plane internal electric field and the potential gradient at the edge. While, the MA cations in noncentrosymmetric structure pointed to the opposite directions to cancel the dipole moments and resulted the potential gradient absent at the edge. This internal electric field had a huge impact on the electronic structure of the 2D perovskites. In the case of *n* ≥ 2, the bandgap of the edge region dramatically decreased compared to that in the bulk region. The narrowed bandgap was the result of the shift of conduction and valence bands, rather than formatting mid-gap defect states (Figures 3C–3E). Besides, the partial charge density calculation showed that the charges were strongly localized at the edges rather than spreading over the entire octahedral layer like that in the bulk region. The localized charges induced by internal electric fields were considered to be the reason for longer emission lifetimes at the edges than that in the bulk. On the contrary, the bandgap alignment of *n* = 1 remains the same through the whole region and the charge densities disperse evenly over the entire inorganic layer due to the lack of the internal electric field. This can explain why edge states only emerged on the *n* ≥ 2 2D perovskites with orientable A-site cation. Moreover, without considering entropy and thermodynamic effects at a limited temperature, the noncentrosymmetric structure was energetically stable for *n* ≥ 2. But in practice, both centrosymmetric and noncentrosymmetric structures of *n* ≥ 2 can exist under room temperature. For *n* = 2, the energy difference between these two structures was smaller than that for *n* > 2. As a result, *n* = 2 could exhibit centrosymmetric structure under room temperature which showed no edge state emissions. Overall, compared to the above lattice mismatch and chemical property theory, the internal electric field theory can well explain the reported experimental observations of the edge states, for example, the absent edge states of *n* = 1 and the inconsistent observations of the edge states for *n* = 2.

Except for theoretical calculation, some experimental results also support that the edge states are the intrinsic property. Wang (Wang et al., 2020) and Feng (Feng et al., 2018) used XRD and transmission electron microscopy selected area electron diffraction (TEM-SAED) to detect the crystal structure of exfoliated crystals and nanowires, respectively. Their result showed that the perovskites samples were pure phase without structure distortion (Figures 3F and 3G). Wang et al. (Wang et al. 2020) also performed a Raman mapping to examine the chemical composition in the edge and bulk regions. These two regions shared the identical Raman spectrum, demonstrating the purity of the crystal edge (Figure 3H). These experimental results ruled out structural distortion of the crystal, assuming that the edge states originated from the intrinsic property of the 2D RP perovskites.

**Figure 4:**
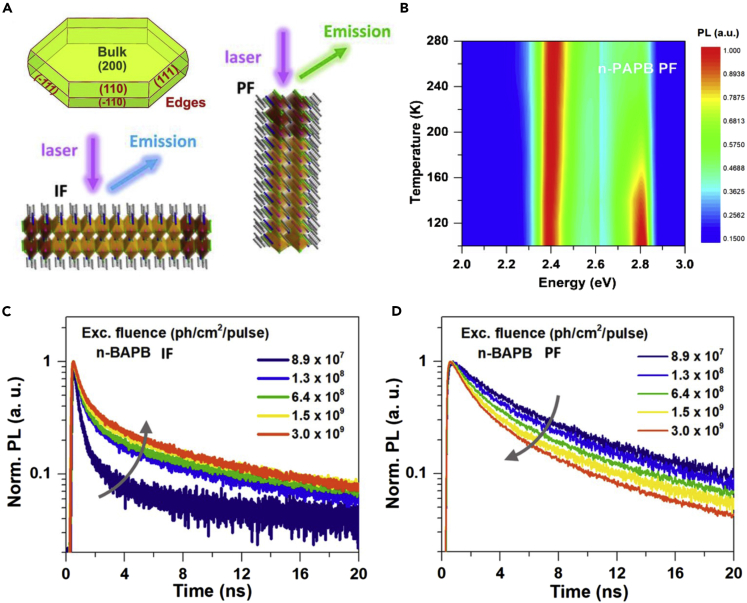
PL experiments by exciting different facets of 2D RP perovskites (A) Macroscopic morphology of a 2D RP perovskite crystal and the schematics of different PL excitation modes. (B) Temperature-dependent PL spectra of (n-PB)_2_(MA)Pb_2_Br_7_ (n-PAPB) by excitation the perpendicular facet (PF). (C and D) Excitation intensity-dependent TR-PL pattern of (n-BA)_2_(MA)Pb_2_Br_7_ (n-BAPB) with different excited facets. Adapted from (Liang et al., 2021). Copyright © 2021, American Chemical Society.

Recently, Liang et al. (Liang et al. 2021) proposed a new hypothesis about the origin of edge states by studying the PL properties excited from different facets of bulk single crystal (perpendicular facet and in-plane facet, denoted as PF and IF, respectively. Figure 4A), and they suggested that the self-trapped exciton was the reason of the edge states. The temperature-dependent PL measurements of (n-PB)_2_(MA)Pb_2_Br_7_ (n-PB is n-pentylamine) showed anomalous behavior. When being excited at the PF of the crystal, it exhibited two PL peaks, the high energy peak originated from band edge recombination and the lower energy peak originated from edge state emission. As temperature decreased, the high-energy PL emission became dominant at cryogenic temperature (Figure 4B), which was against the traditional exciton trapping mechanism (Liang et al., 2021). As a result, they assigned the low energy edge state emission to the self-trapped exciton formed by Fröhlich interaction, which would decrease at low temperature (Wright et al., 2016). Besides, the time-resolved PL measurements also demonstrated the different PL dynamics between PF and IF (Figures 4C and 4D), where the PL lifetime of IF increased with the increasing excitation intensity while the PL lifetime of PF decreased with the increasing excitation intensity. The former can be explained by trap saturation model (Lu et al., 2021; Stranks et al., 2014; Wen et al., 2016; Zheng et al., 2016) and the latter can be interpreted by the self-trapped exciton model. The radiative recombination of self-trapped exciton can be the main depopulation pathway of PF. At high excitation intensity, the self-trapped exciton may have a higher mobility and be easier to be trapped by the defect states, leading to a faster PL decay.

### The extrinsic property theory

Although the intrinsic property theory based on the DFT calculation can suitably explain some parts of the experimental phenomena, there are still some phenomena that cannot be interpreted by the intrinsic property theory. For example, low energy emissions can be controlled by external conditions (Shi et al., 2019; Zhao et al., 2019). Besides, some TEM-SAED results observed the structure distortion at the edge region of 2D RP perovskites (Shi et al., 2019; Zhao et al., 2019), which contradicts that of clean edge observations, pointing out the existence of structure distortion at the crystal edge. These results imply that the intrinsic electronic properties may not be the only reason to determine the edge states; the structure distortion at the edge also plays an important role and therefore the extrinsic influence is not negligible.

**Figure 5:**
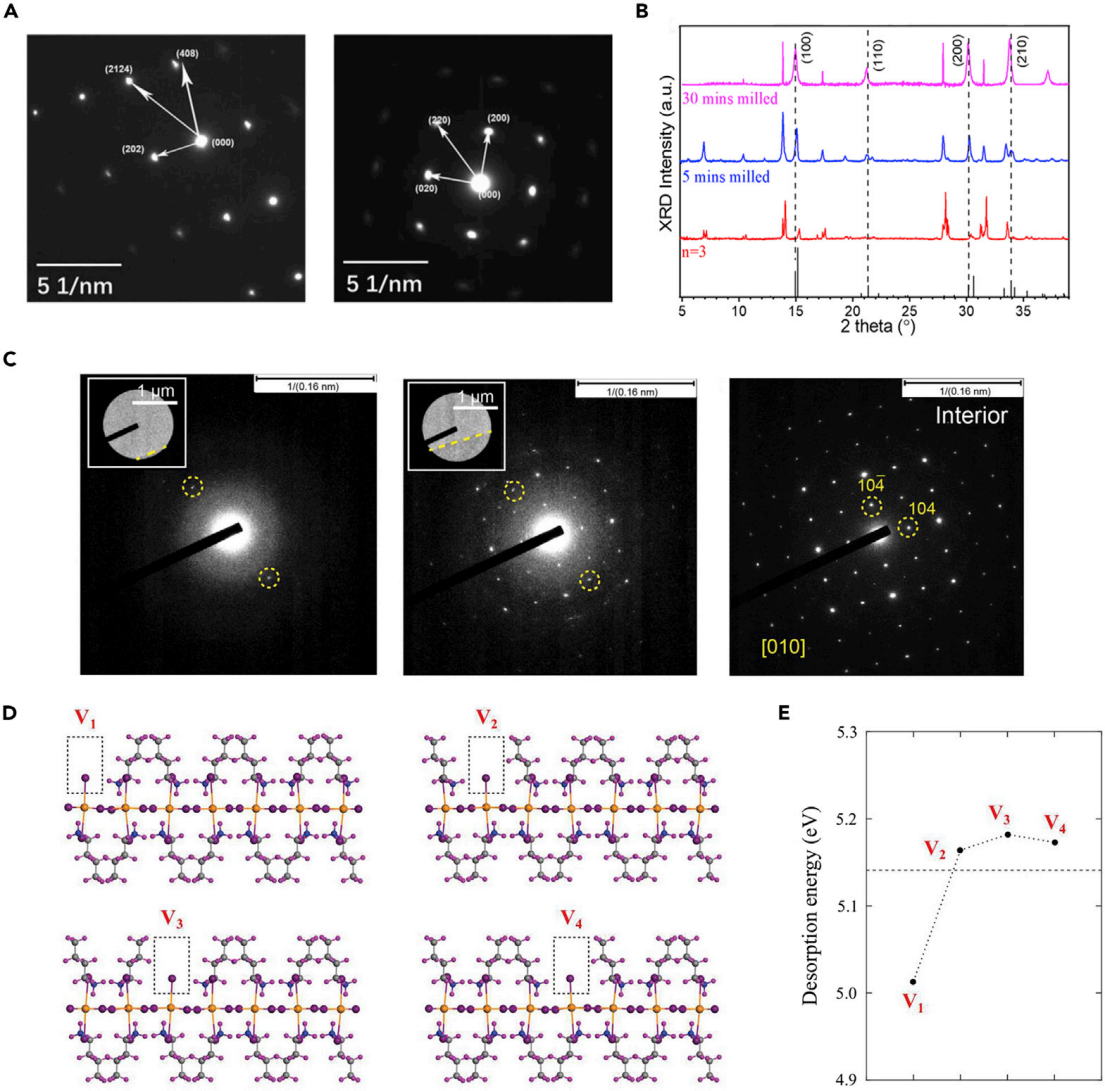
Characterization and DFT calculation of the structure distortion of 2D RP perovskites (A) SAED patterns of (BA)_2_(MA)_2_Pb_3_I_10_ (*n* = 3) exfoliated 2D perovskite crystal. Left panel: collected from the interior region. Right panel collected from the crystal edge region, Reprinted from (Zhao et al., 2019). © 2019, American Chemical Society. (B) Evolution of XRD of (BA)_2_(MA)_2_Pb_3_I_10_ before and after freeze milling for 5 and 30 min, Reprinted from (Qin et al., 2020). Copyright © 2020, American Chemical Society. (C) Evolution of SAED patterns of BA_2_FAPb_2_I_7_ exfoliated crystal edge with the selected area moving to the interior areas gradually. Reprinted from (Shi et al., 2019). Copyright © 2019, American Chemical Society. (D) Relaxed structures of BA_2_PbI_4_ nanoribbon containing organic vacancies V_i_ at different positions along its width, where i = 1, 2, 3, 4. V_1_ is located at the free edge, while V_4_ is situated in the central region of the nanoribbon. (E) Desorption energy of the organic molecule at the various vacancy sites. Figure 5D and 5E reprinted from (Kripalani et al., 2022) Copyright © 2022, American Chemical Society.

As mentioned above, Zhao et al. found that the edge state emission of 2D RP perovskites can be generated or eliminated by MA^+^ or BA^+^ solution treatment, respectively (Zhao et al., 2019). When they further performed TEM-SAED to detect the crystal structure of the edge, it was found that some sites exhibit a different crystal structure from the bulk crystal structure of (BA)_2_(MA)_2_Pb_3_I_10_ (*n* = 3). Such specific structure in the edge matches with that of the 3D perovskite (MAPbI_3_), indicating the existence of 3D perovskite structure at the edge (Figure 5A). It is worth noting that many exfoliated samples with *n* ≥ 2 were found to have no edge state emission, but the edge state emission can be generated by the treatment of MA cation exchange. As a consequence, it was suggested that the edge state emission of exfoliated 2D perovskite crystal was the result of the formation of 3D structure at the edge because of the lost BA cation.

The existence of a 3D perovskite structure at the edge was also supported by Qin et al. (Qin et al. 2020); they pointed out that the trace amount of structure distortion at the edge region is difficult for XRD to detect and identify due to the limited sensitivity. Thus, freeze milling was applied to form a smaller crystal, increasing the edge-to-bulk ratio as well as the sensitivity of XRD. Moreover, milling under low temperatures (77 K) will protect the crystal from chemical or composition variation. After 30 min freeze milling, the characteristic diffraction peak of 3D perovskites appeared on the XRD pattern of the samples, which further confirmed the existence of 3D perovskites structure at the edge region (Figure 5B).

However, there are different opinions about the nature of the structure distortion at the edge; instead, it was suggested that structure distortion at the edge is not 3D perovskites. Shi et al. reported that edge state emission of 2D RP perovskites can be induced by exposure to moisture. They proposed that with the assistance of water molecules the adjacent layers would partially merge, leading to the formation of edge states (Shi et al., 2019). To further confirm this supposed mechanism, TEM-SAED patterns were performed on the BA_2_FAPb_2_I_7_ sample after 2 h of exposure to air. New diffraction spots emerge at the edge region which were different from those in the bulk regions. The emerged diffraction spots cannot be attributed to the lattice plane of BA_2_FAPb_2_I_7_, FAPbI_3_, PbI_2_, or Pb (Figure 5C), which indicated the edge region underwent an atoms rearrangement and formed a new crystal structure rather than a 3D perovskite structure.

Kinigstein et al. (Kinigstein et al. 2020) performed the grazing incidence wide-angle scattering (GIWAXS) to study the edge state structure located at the surface of perovskite films. Their result indicated the edge states exhibited a long-range (∼1 nm) structure distortion and were highly oriented in an out-of-plane direction. They could not identify the actual structure of this distortion because of the close similarity of the inorganic sublattice in the 3D and 2D RP crystals. The possibility of the lattice distortion originating from the 3D phase could not be completely ruled out.

Recently, Kripalani et al. performed a DFT study based on the nanoribbons of BA_2_PbI_4_ (Kripalani et al., 2022). They found that the band alignment becomes narrower and narrower with increasing the width of nanoribbons, which is ascribed to the structure distortion of the inorganic octahedron at the edge due to the strong tensile stresses. The stress-induced structure distortion was supposed to be the reason for the 2D RP perovskite edge states. What’s more, the stress at the edge makes 2D RP perovskite unstable and 2D RP perovskites exhibit a tendency to release the stress by structural reorganization, like forming BA-vacancies or substitution. The desorption energy of the different vacancy sites was also calculated based on the BA_2_PbI_4_ nanoribbon with seven inorganic octahedrons. As shown in Figures 5D and 5E, the BA-vacancy V_1_ located at the edge of the nanoribbon has the lowest desorption energy (5.01 eV) compared to that of the other vacancy sites (5.16–5.18 eV), indicating the BA-vacancy is more likely to occur at the edge of 2D RP perovskites. The desorption energy of the BA_2_PbI_4_ nanosheet (with infinite inorganic octahedrons) was calculated as 5.14 eV (dash line), which is similar to the vacancies that lie in the inner part of the nanoribbon, implying that BA-vacancy trends to form in the edge of the practical crystal. This calculation indicated the structure instability of 2D RP perovskite is induced by edge stress, which agrees with the former observation of the loss BA cation at the edge and the reaction with other molecules like moisture or MA cation (Shi et al., 2019; Zhao et al., 2019).

## Discussion and prospective

According to the above discussion, we can draw a basic picture of the edge states of 2D perovskites, a narrower band gap indeed exists at the edge of 2D RP perovskites which is supported by DFT calculation (Hong et al., 2021; Kepenekian et al., 2018; Kripalani et al., 2022; Wang et al., 2020). However, these intrinsic theories fail to explain the phenomenon that edge state emission of the perovskites with *n* ≥ 2 can be induced by moisture or MAI solution rinsing process, which inferred that the band gap is not the only factor for the edge states emission. Instead, we deduce that the structure distortion is the reason for the origin of the edge state because it well explains the edge state emission of the perovskites with *n* ≥ 2 induced by moisture or MAI solution rinsing process; and it also explains why the low energy emission is only observed in some spots of the crystal edge instead of the entire edge of the crystal. However, the detailed formation process of structure distortion is still unclear and the proposed atomic arrangements of distortion are controversial. This is expected to become a key research topic in the future. Besides, comprehensive knowledge of how the structure distortion affects the electronic structure and other physical properties of 2D RP perovskites is not available; this can be a crucial point to have a thorough understanding of 2D RP perovskite edge states.

The edge state emission of 2D RP perovskites with *n* = 2 is very inconsistent and therefore it is highly debated if there is an effect of the edge states for 2D RP perovskite for *n* = 2. When Blancon et al. first reported the edge states of 2D RP perovskites (Blancon et al., 2017), they observed that BA_2_MAPb_2_I_7_ did not have edge state emission, suggested that 2D RP perovskites with *n* = 2 have no edge states, and proposed a lattice mismatch model to support their view (Kepenekian et al., 2018). The absent edge state emission of BA_2_MAPb_2_I_7_ was also observed by the latter reports (Feng et al., 2018; Shi et al., 2019). However, Zhao et al. (Zhao et al. 2019) observed the opposite experimental phenomenon that the pristine BA_2_MAPb_2_I_7_ exhibited the edge state emission and they proposed that it can be ascribed to the structure distortion at the edge. Later, another theory was proposed to explain this contradictory phenomenon (Hong et al., 2021), that was BA_2_MAPb_2_I_7_ has noncentrosymmetric and centrosymmetric structure under room temperature and the former structure has edge state emission while the latter did not. Besides, when the A-site cation changed from MA^+^ to FA^+^ or the ligand changed from BA^+^ to ThMA^+^(2-thiophenemethylamine cation), the edge state emission was observed in the 2D RP perovskites with *n* = 2 (Shi et al., 2019; Zhao et al., 2020). Recently, by combining scanning tunneling microscopy and scanning tunneling spectroscopy, Shih et al. (Shih et al. 2021) observed different electronic structures between BA_2_PbI_4_ (*n* = 1) and BA_2_MAPb_2_I_7_ (*n* = 2). An extra energy state is observed near the valance band of n = 2 perovskites within the bandgap, and they considered that the extra energy state was the edge state that led to the low energy emission of 2D RP perovskites. According to the above discussion, 2D perovskites with *n* = 2 (BA_2_MAPb_2_I_7_) have edge states. The edge state emission may not be observed in some cases; one possible reason is that the edge state emission is hard to observe in the macroscopic experiment because it only appears at the edge region and it is relatively weak because there exists defect trapping at the edge. It is generally accepted that defect trapping exists at the edge of 2D perovskites, particularly for unpassivated samples, for example, mechanically exfoliated platelets. Based on this feature, our group developed a method for measuring the exciton in-plane diffusion in 2D RP perovskite of *n* = 1 and determining diffusion length of 1.8 μm, using separated excitation-detection spot of micro-TRPL technique, in which the defect trapping acted as quenching source in the edge (Zhou et al., 2020).

In addition to the origin of the edge states in 2D RP perovskites, an in-depth understanding of how the edge states affect the optoelectronic properties is another important task. Recently, we revealed that the (BA)_2_(MA)_n-1_Pb_n_I_3n+1_ single crystal with layer number *n* ≥ 2 exhibited anomalous free carrier behavior even though the binding energy was more than 100 meV, while their *n* = 1 counterpart exhibited exciton behavior (Lu et al., 2021). We assigned the different carrier dynamic properties to the efficient exciton dissociation effect of the edge states. After the exciton is photogenerated in the bulk region, it would undergo efficient in-plane exciton diffusion (Deng et al., 2020; Seitz et al., 2020; Zhou et al., 2020; Ziegler et al., 2020) to the edge region. For the perovskites with *n* ≥ 2, the exciton would be dissociated into free carrier through the edge states and exhibited the free carrier behavior. But for the *n* = 1 perovskites, due to lack of edge states, the exciton at the edge would not be dissociated and exhibit the exciton behavior. Besides, the exciton-phonon coupling strength at the edge region was found to be stronger than the bulk region (Liang et al., 2021). Except for carrier property and exciton-phonon coupling, 2D RP perovskites have many other novel optoelectronic properties like giant Rashba splitting, optical nonlinearities, stark effect, and so on (Gan et al., 2021). All of these properties are most likely affected by the edge states in some extent; further investigation and deep understanding are nevertheless demanded.

The conductivity is considered to be another unique property of the 2D RP perovskites intimately relevant to the edge states. However, the observations based on c-AFM and CGM are quite different than the spectroscopic observations. The edge states are consistently considered to be absent in 2D RP perovskite of *n* = 1. While, the c-AFM and CGM observations showed that *n* = 1 2D RP perovskite exhibited edge state conductivity (Wang et al., 2019b, 2020). The DFT calculation also suggested that the higher charge density existed in the edge region for *n* = 1 (Wang et al., 2020), proposing the existence of edge states in *n* = 1. It is noteworthy that CGM and c-AFM are the contact characterization techniques while spectroscopic characterization is a non-contact technique. Such difference between these characterization techniques is probably the reason for the different observation results. But right now, there is still a lack of reasonable explanation for the contradictory observations.

In summary, the edge states of the 2D RP perovskites exhibit unique properties, like super-ability of exciton dissociation, low-energy PL emission, longer carrier lifetime at the edge, and high conductivity. It has been confirmed that the edge states can significantly impact the performance of 2D RP perovskite-based optoelectronic devices. The edge states dissociate efficiently excitons into free carriers, and the free carriers are easier to be extracted by the devices, leading to the improved performance of solar cells and photodetectors (Blancon et al., 2017; Feng et al., 2018; Zhao et al., 2020). It is noteworthy that when discussing the solar cells based on 2D RP perovskites with *n* ≥ 2, the carrier transfer between multi-phase cannot be ignored because the mixed multi-phases usually exist in the 2D RP perovskite films with *n* ≥ 2. The carrier transfer between different phases in films could also lead to a longer carrier lifetime and low energy emission (Liu et al., 2017). When discussing the edge state of 2D RP perovskite films with *n* ≥ 2, it should be ensured that the 2D RP perovskite films are pure phase. However, for the applications like LED and laser, the free carriers are harmful to the devices because they reduce the radiative recombination. As a result, it is essential to take into account the effect of the edge states when designing the optoelectronic devices of 2D RP perovskites.

To date, the understanding of the edge states in 2D RP perovskites is insufficient and many questions remain unsolved, in particular, the accurate atom arrangement on the edge region and the controversial observation result on *n* = 1. Although there have been many reports on the edge states, a consensus is not yet reached. What is more, the properties like carrier dynamics and exciton-phonon coupling of 2D RP perovskites are strongly related to the edge states. This article aims at providing a critical review to promote thorough investigations on the edge states and their impact to enable broad applications for future high-performance optoelectronic devices.
